# Strategies for coping with family members of patients with mental disorders

**DOI:** 10.1590/1518-8345.1311.2799

**Published:** 2016-09-09

**Authors:** Daniele Alcalá Pompeo, Arélica de Carvalho, Aline Morgado Olive, Maria da Graça Girade Souza, Sueli Aparecida Frari Galera

**Affiliations:** 1 PhD, Adjunct Professor, Faculdade de Medicina de São José do Rio Preto, São José do Rio Preto, SP, Brazil.; 2 Undegraduate student in Nursing, Faculdade de Medicina de São José do Rio Preto, São José do Rio Preto, SP, Brazil. Scholarship holder of the Scientific Initiation Program at the Conselho Nacional de Desenvolvimento Científico e Tecnológico (CNPq), Brazil.; 3 RN, student of the Obstetric Nursing of the Residency Program, Escola de Enfermagem, Universidade de São Paulo, São Paulo, SP, Brazil.; 4 PhD, Associate Professor, Escola de Enfermagem de Ribeirão Preto, Universidade de São Paulo, PAHO/WHO Collaborating Centre for Nursing Research Development, Ribeirão Preto, SP, Brazil.

**Keywords:** Adaptation, Psychological, Psychiatric Nursing, Mental Disorders, Family Health

## Abstract

**Objective:**

to identify the coping strategies of family members of patients with mental disorders and relate them to family member sociodemographic variables and to the patient's clinical variables.

**Method:**

this was a descriptive study conducted at a psychiatric hospital in the interior of the state of São Paulo, with 40 family members of hospitalized patients over the age of 18, and who followed the patient before and during hospitalization. We used tools to characterize the subjects and the Folkman and Lazarus Inventory of Coping Strategies.

**Results:**

the coping strategies most often used by family members were social support and problem solving. Mothers and fathers used more functional strategies (self-control p=0.037, positive reappraisal p=0.037, and social support p=0,021). We found no significant differences between the strategies and other variables examined.

**Conclusion:**

despite the suffering resulting from the illness of a dear one, family members make more use of functional strategies, allowing them to cope with adversities in a more well-adjusted way.

## Introduction

Mental illness is currently a major topic worldwide, as it is increasingly found in the day-to-day life of the population. About 700 million people worldwide suffer some form of mental or neurological disorder. One out every four persons will develop some form of these disorders during his or her lifetime. It is unusual to find a family that does not have at least one member suffering from some form of mental disorders^1^. This high incidence is directly related to the use of drug and alcohol, and to the modern life-style, where people are more exposed to stressors^1^.

We often find that not only the individual involved suffers losses resulting from this situation, but also their family members and society at large. The family experiences a sequence of stressors that interfere in family unity, such as the diagnosis of the disease itself, the adverse effects of medication, the individual's inability to perform daily tasks, possible changes in economic and social status, uncertainty as to whether there is a cure, and the possibility that the disease may become chronic^2^ ^-^ ^3^. 

Stressors are coped with based on how significant they are for those involved. Coping means trying to overcome that which is causing stress, and may refocus the significance associated with the difficulties, guide the individual's life and keep him/her physically, psychologically and socially healthy^4^.

The literature mentions numerous studies on coping strategies for family members and caregivers of people with chronic diseases^5^ ^-^ ^6^. Some studies describe these strategies among the caregivers of patients with schizophrenia and psychotic diseases, however using scales that have not been validated in Portuguese^3^ ^,^ ^7^ ^-^ ^8^. Other studies reveal the overload and poor quality of life of the caregivers of patients with psychiatric disorders^9^ ^-^ ^10^. However, literature on family member coping in the case of people with mental disorders is scarce.

Families are generally vulnerable and unprepared to cope with the entire process of the illness and treatment^3^ ^,^ ^8^. For this reason, nurses and other healthcare professionals who live with this reality have a fundamental role to play on the patient/family binomial, supporting them and helping them identify stressors, understanding and recognizing how they cope with problems so as to intervene and minimize suffering, thus making a positive contribution to their readjustment^2^ ^,^ ^7^ ^,^ ^10^.

Thus the objectives of this study were to identify the coping strategies of the family members of patients with mental disorders hospitalized in a psychiatric hospital, and associate them with family member sociodemographic variables and patient clinical variables.

## Method

A descriptive, exploratory cross-sectional study performed at a 250-bed psychiatric hospital in the interior of São Paulo working with SUS, healthcare plan and private patients, thus serving a heterogeneous group of people in terms of socioeconomic and cultural level. 

The study population was made up of the family members of patients hospitalized in said hospital due to mental disorders between October and December 2013. The inclusion criteria were: 18 years or older, be related to the hospitalized patient in some way, and have followed the patient before and during hospitalization. Where two or more family members qualified, we chose the one spending more time caring for the hospitalized patient (indicated by the family members themselves), resulting in a sample of 40 subjects. 

The size of the sample was limited by the time available for data collection (October 1 - December 31 2013), which in turn was a function of the human resources and time available for this study. 

Data collection used two tools: sociodemographic description of the family members and clinical status of the patient, and the Folkman and Lazarus *Inventory of Coping Strategies* (FLICS)^4^. 

The Folkman and Lazarus Inventory of Coping Strategies includes the thoughts and actions people used to handle the internal or external demands of a specific stressful event. It is a list of 66 items answered using a Likert-type scale, with four possible answers (0: never used this strategy; 1: used it a bit; 2: used it a lot; 3: used it extensively). This scale is not associated with a total score as a sum for assessment, as the items should be assessed using average scores within each factor^4^.

This tool was translated and validated for Brazilian Portuguese, demonstrating correspondence between the original English language version and the translated one, allowing it to be applied to other studies. In the original study, Cronbach's alpha ranged from 0.56 to 0.85 across the factors. The items that make up this tool are split into eight factors: comfort, distance, self-control, social support, acceptance of responsibilities, escape-avoidance, problem solving and positive reappraisal ^11^. 

The elements of FLICS are split into two categories: (1) functional strategies, made up of self-control, social support, problem solving, positive reappraisal and acceptance of responsibilities, and (2) dysfunctional strategies, corresponding to confrontation, distancing and escape and avoidance^12^.

We ran a pre-test with five subjects to test, adjust, fine-tune and measure duration of application of the proposed data collection tools, which did not result in any changes. Data was collected during verbal interviews conducted during visiting hours.

The data was processed and analyzed using *Statistical Package for Social Science* (SPSS) version 19 *for Windows, IBM Company, Copyright* 2010. For descriptive data analyses we used position measurements and variability for continuous variables, and simple frequency for the categorical variables. 

To assess the coping strategies we calculated the mean, median and average deviation of the score obtained for each factor. Mean scores were calculated based on the number of items in each factor. The internal consistency of FLICS data was checked using Cronbach's alpha. 

To analyze the mean scores of the association between coping strategies and sociodemographic variables, we used Mann-Whitney's non-parametric test to compare the two groups, and the Kruskal-Wallis test and Dunn's post-hoc test to compare more than two groups. We used a significance level of 0.05.

This study complies with Brazilian and international standards of ethics for research involving human beings, and received a favorable opinion from the local Ethics Committee (Document # 300,424). All of the patients signed the Free and Informed Consent Form.

## Results

Of the 40 family members of patients with mental disorders, 20 were male and 20 female, with ages ranging from 18 to 67, with an average of 39 and SD of 14.7 years. Regarding the family relationship, seven (17.5%) were a parents (father or mother), 10 (25%) were the children, nine (22.5%) a sibling, and 14 (35.0%) were a nice/nephew, grandchild, in-law or cousin. 24 (60%) of the participants had ten or more years of schooling, and half (50%) of the sample claimed to be Roman Catholic.

Regarding patient clinical variables, the most frequent was bipolar affective disorder (n=16; 40.0%), followed by depression (n=13; 32.5%) and schizophrenia (n=11; 27.5%). Most (n=26 65.0%) have had the disease for over 10 years, many with recurring delirium (n=17; 42.5%) and hallucinations (n=20; 50.0%).

The coping strategy most often used by family members was social support, and the least used was confrontation. Functional strategies were the ones used most often. The internal consistency of FLICS factors measured using Cronbach's alpha ranged from 0.44 to 0.79 (Table 1).

**Table 1:** Mean scores of the coping strategies reported by family members of patients with mental disorders. São José do Rio Preto, SP, Brazil, 2013

Coping strategy	Mean score	Standard deviation	Median	Cronbach's alpha
Confrontation	0.69	0.92	0.50	0.44
Distancing	0.83	1.03	0.35	0.62
Self-control	1.10	1.10	0.80	0.47
Social support	1.66	1.09	1.66	0.66
Acceptance of responsibilities	1.09	1.02	0.85	0.70
Escape-avoidance	1.25	1.13	1.0	0.78
Problem solving	1.57	1.06	1.75	0.57
Positive reappraisal	1.42	1.14	1.44	0.79

There was a significant association between family relationship and self-control strategies (p=0.037), social support (p=0.021) and positive reappraisal (p=0.037), indicating that parents (mother and father) use this strategy more often than children, siblings or other family members (Table 2).

**Table 2:** Mean values of coping strategies mentioned by the family members of patients with mental disorders by sociodemographic variable. São José do Rio Preto, SP, Brazil, 2013

Family member sociodemographic variables		Mean value of the factors in the Folkman and Lazarus Inventory of Coping Strategies							
		Confron-tation	Distancing	Self-control	Social support	Acceptance of responsibilities	Escape-avoi-dance	Problem solving	Positive reappraisal
Gender									
	Male	0.72	0.75	1.01	1.53	1.00	0.95	1.37	1.48
	Female	0.77	0.93	1.19	1.78	1.16	1.55	1.81	1.33
	p-value*	0.903	0.310	0.391	0.349	0.355	0.093	0.093	0.532
Age group									
	18 - 30	0.70	0.74	0.93	1.56	0.91	1.18	1.51	1.35
	31 - 59	0.67	0.84	1.19	1.66	1.17	1.17	1.67	1.42
	60 or over	1.29	1.24	1.30	1.99	1.35	1.87	1.50	1.55
	p-value^†^	0.143	0.283	0.512	0.452	0.294	0.376	0.797	0.782
Years of schooling									
	0 to 4	0.80	1.18	1.20	1.60	0.97	1.16	1.41	1.03
	5 to 9	0.68	0.65	0.90	1.66	0.99	1.40	1.57	1.34
	10 or more	0.76	0.83	1.15	1.66	1.15	1.20	1.64	1.53
	p-value^†^	0.865	0.238	0.380	0.917	0.790	0.749	0.638	0.225
Income									
	1 - 3	0.71	0.83	1.08	1.51	1.03	1.30	1.43	1.22
	4 - 7	0.83	0.93	1.05	2.02	1.24	1.21	1.96	1.86
	Does not know	0.79	0.76	1.28	1.93	1.13	1.00	1.95	1.81
	p-value^†^	0.773	0.897	0.770	0.132	0.691	0.737	0.108	0.069
Has partner									
	Yes	0.72	0.80	1.18	1.76	1.17	0.93	1.79	1.58
	No	0.77	0.88	1.00	1.52	0.98	1.64	1.34	1.19
	p-value*	0.611	0.622	0.502	0.539	0.234	0.056	0.055	0.134
Relationship with patient^‡^	
	Father or mother	1.02	1.05	1.71^§^	2.21^§^	1.32	0.93	1.96	1.91^§^
	Son/Daughter	0.69	0.69	1.18^§||^	1.86^§||^	1.36	1.85	1.75	1.65^§||^
	Sibling	0.66	1.02	0.89^||^	1.27^||^	0.72	1.11	1.50	0.94^||^
	Other	0.70	0.72	0.87^§||^	1.47^§||^	0.99	1.07	1.35	1.28^§||^
	p-value^†^	0.646	0.406	0.037	0.021	0.089	0.137	0.283	0.037

*Mann-Whitney* *p-value *(<0.05); Kruskal-Wallis with Dunn Multiple Comparison test* †p-value (P<0.05); ‡Different symbols in same column v differ significantly according to Dunn's Multiple Comparison Test (P<0.05) (§significant difference between || and §|| no significant difference between § and ||)

Women tend to escape and avoidance and problem solving more than men. Family members earning between four and seven minimum salaries are more prone to using positive reappraisal. 

We did not find significant differences in coping strategies in terms of age, years of schooling or religion, but did find a trend towards a significant difference for the variable "has partner'. Family members reporting no partner are more likely to use escape and avoidance for coping with their problems, while those who claim to have a partner score more highly in problem solving.

The only clinical variable with marginally significant results was the presence of psychotic symptoms, showing that problem solving tends to be more used by family members of patients who do not display psychotic symptoms (Table 3).

**Table 3:** Mean values for FLICS factors among the caregivers of patients with mental disorders according to the clinical variables of the patient. São José do Rio Preto, SP, Brazil, 2013

Patient clinical variables		Mean value of the factors in the Folkman and Lazarus Inventory of Coping Strategies							
		Confron-tation	Distancing	Self-control	Social support	Acceptance of responsibilities	Escape-avoidance	Problem solving	Positive reappraisal
Patient disorder									
	Schizophrenia	0.72	0.82	1.18	1.64	1.11	1.09	1.38	1.26
	ABD	0.81	0.92	1.12	1.72	1.13	1.43	1.68	1.58
	Depression	0.69	0.75	1.00	1.57	1.00	1.15	1.65	1.32
	p-value*	0.744	0.734	0.833	0.695	0.769	0.668	0.600	0.423
Disease duration									
	30 days or less	0.66	0.35	0.80	1.33	0.67	1.00	1.31	1.36
	31 days - 3 years:	0.96	1.02	1.40	2.10	1.11	1.41	1.62	1.49
	4 to 10 years	0.58	0.85	1.45	1.78	1.49	0.75	1.75	1.57
	11 or more years	0.73	0.87	1.02	1.58	1.08	1.32	1.60	1.37
	p-value*	0.595	0.278	0.371	0.402	0.193	0.409	0.955	0.995
Psychotic symptoms												
	Yes	0.77	0.89	1.14	1.56	1.01	1.50	1.37	1.38
	No	0.71	0.78	1.05	1.75	1.17	0.97	1.84	1.44
	p-value^†^	0.631	0.594	0.733	0.480	0.513	0.095	0.052	0.828

*Value of P for the Kruskal-Wallis test with Dunn's Multiple Comparison Test (P<0.05); *^†^* Value of P for the Mann-Whitney Test (P<0.05).

Family member coping strategies differ by disease type and duration. However, we found no significant differences between coping strategies and patient clinical variables (Table 3). 

## Discussion

The results of this study show that the family members of people with mental disorders preferentially use social support, problem solving and positive reappraisal to care for their ill family member. Fathers and mothers mention using more self-control (p=0.037), positive reappraisal (p=0.037) and social support (p=0,021). These strategies are classified as functional and constitute positive ways of coping with problems^12^. Adopting these strategies may contribute to reducing the stress and overload of caregivers ^13^.

According to the Lazarus and Folkman perspective of coping with stress, social support is based on the person's effort to seek support within his/her social, professional and emotional spheres^14^. Studies in the United States^13^, India^3^ ^,^ ^15^ and China^16^ ^-^ ^17^ ^)^ found that the search for social support is an important strategy for caring for a mentally ill family member. Family, friends, religions, doctor visits and other resources provided by the healthcare system and the community were the forms of social support most often used by the subjects^3^ ^,^ ^13^ ^,^ ^15^.

Educating caregivers about social support was effective in reducing stress among those caring for patients with dementia, and family members participating in interventions to build this skill developed fewer symptoms of depression and were more satisfied with their life^16^.

Positive reappraisal describes efforts to create significant meaning in the face of problems, in order to provide personal growth^14^. Control over emotions related to sadness serves as a way to assign new meaning, learn and change from a conflicting situation^18^. A study in the United States revealed a number of family reflection as examples of positive reappraisal*. I must grow as a person in light of such difficulties. I must look at the problem differently, from a more positive perspective. I am looking for something god about what I am experiencing. I always learn something from experience(* *^13^* *^_)._^* People with higher incomes tend to use this strategy, which may be associated with increased years of schooling and the positive influence this has on coping with a stressful situation^19^.

Although we have been unable to establish a positive correlation with the variables investigated, problem solving is one of the three strategies most often used by the participants in this study. It involves changing a situation through detailed, critical analysis to get satisfactory outcomes for problem solving ^14^. Rather than deny or avoid a stressful day-to-day situation, the person opts to solve problems and change mindsets so that he or she is able to cope with normal pressures, reducing or even eliminating situations that cause stress^18^. 

Self-control refers to the efforts made by a person to control his/her feelings or actions, in the face of stressful stimuli^14^. To deploy this strategy a person must be aware of and understand his/her emotions, and be able to manage his/her behavior^14^. 

A study in Ireland to find ways of coping with a family member with a mental disorder showed that caregivers more often used social support and self-control, both variables associated with being male and younger^20^.

Functional strategies may be hard for family members who are vulnerable and suffering to deploy, especially in the early stages following the diagnostic, as the family is still adjusting to change. Here support groups may be effective strategies to help family members through this process, making it easier for them to identify the problem, express their feelings and learn from others, and in these situations information and support are shared, hope is given and similar problems and difficulties are resolved. These groups also reduce social isolation, stigma and bias^21^.

A study of caregivers of elderly patients with dementia highlighted functional strategies as addressable protection factors for reducing stress, after they received three-months training in psychosocial skills. This educational problem also helped caregivers seek more social support, further using problem solving ^16^.

Psycho-educational training of the family members of patients with mental disorders have been shown in the literature to be effective ways to promote positive coping with the problems, in particular: group training^16^, using manuals/handbooks^22^, using the telephone^23^ ^-^ ^24^ or online resources^25^.

Survey participants who were the sons or daughters of people with mental disorders had higher scores in the escape and avoidance dysfunctional strategy. These results suggest that the sons and daughters of patients with mental disorders are aware that they can help care for their parent, but at the same time want to avoid the situation, in an attempt to make it go away. They are unable to overcome the suffering, thus taking on the role of caregiver. 

Another study found that distancing, escape and avoidance were strategies often used by younger family caregivers living with the mentally ill patient, fostering the continuation of fears, concerns and physical and psychological problems^20^.

Individuals with fewer years of schooling often use dysfunctional strategies, as it is hard for them to find the source of the problems and options to solve or address it^19^. In this survey, family members with fewer years of schooling had higher scores in confrontation compared to those with more years of schooling. 

Confrontation is an aggressive manner chosen to change a situation, such as anger and inflexibility^14^. This may be the result of constant periods of crisis and instability resulting from the disease, as well of moments of suffering and uncertainty regarding the future and social and financial concerns and difficulties^2^.

Individuals reporting that they have partners were more likely to use problem solving, and those without were more likely to use escape and avoidance. Ideally, a partner is associated with affection, daily living, the exchange of experiences and mutual support, which may explain the choice of functional strategy.

The trend to using the functional strategy problem solving among family caregivers of patients who do not display psychotic manifestations may be the result of a more stable clinical situations, and the absence of aggression secondary to delirium and hallucinations. A study of family members of patients with psychotic disorders found that escape, distancing and rumination were often found strategies, all of which are related to the existence of psychological stress^8^.

When assessing reliability, Cronbach's alpha varied from 0.44 to 0.79, showing moderate reliability. There are numerous factors that can influence the reliability of a tool, such as number of items and size of sample^18^. The reliability results of this study are comparable to other studies using the Folkman and Lazarus Inventory of Coping Strategies^11^ ^,^ ^18^.

Furthermore, when applying the *Inventory of Coping Strategies*, we had problems selecting the value to assign to the strategies. It is felt that people need more time to reflect on the items and decide which answer best represents their actions/feelings regarding the conflicts they experience.

After the initial impact of discovering the disease, the family starts to adjust to the new reality. Each moment has meaning, a need, a feeling, ways of coping and specific support networks. Some feelings permeate everything, but their meaning and intensity vary depending on the phase of the adjustment process^2^, which may explain a greater search for functional strategies by family members who have lived with the disease for many years. 

Nurses and healthcare professionals must be in direct contact with the family universe to identify the complex and conflicting realities of these people. This requires planning so that they may provide care and support, including assessment and follow-up of the families involved to learn of their physical, emotional and social difficulties, as well as early perception and recognition of problems or suffering that could change the family dynamic and influence patient treatment. 

It is extremely important to expand studies focused on this theme, always looking to contribute with new perspectives of psychosocial care for the families, with frequent reflections on professional practices and new public policy guidelines, in particular in the areas of mental health and psychiatry.

## Conclusion

The coping strategies most often used by the family members of people with mental and psychiatric disorders are social support and problem solving. Parents use more functional strategies for caring with a child with a mental disorder.

There are few studies to date, either in Brazil or abroad, on the experience of family members of people with mental or psychiatric disorders, and how they cope with the stressors, in particular using a specific theoretical reverence such as the Inventory of Coping Strategies. 

The results of this study are limited because it used a small, non-probabilistic sample, leading to a possible bias and being less representative of the population. We also highlight the fact that we did not look at other variables that may have interfered in the coping strategies and provide better subsidies for our results, such as the persons' personality, overload, social support and stress levels.

However, the results obtained enable understanding how family members face the presence of mental disorder in a dear one, and allows nurses to reinforce the concept that the family is an important care group, thus expanding their scope of action, with interventions designed to manage caregiver overload so that he or she may be able to better deal with the patient's disease, and maintain his/her balance and positive outlook, ultimately impacting the care of the person with mental illness. Furthermore, it makes way for intervention studies to explain new scientific evidence for implementing effective psychoeducation programs for training in these functional skills.
